# Investigating the crosstalk between chronic stress and immune cells: implications for enhanced cancer therapy

**DOI:** 10.3389/fnins.2023.1321176

**Published:** 2023-11-28

**Authors:** YongRong Lei, Fenghui Liao, YiChen Tian, YaNi Wang, Feng Xia, JianHua Wang

**Affiliations:** ^1^Key Laboratory of Biorheological Science and Technology (Ministry of Education), College of Bioengineering, Chongqing University, Chongqing, China; ^2^Key Laboratory of Hepatobiliary and Pancreatic Surgery, Institute of Hepatobiliary Surgery, Southwest Hospital, Third Military Medical University (Army Medical University), Chongqing, China

**Keywords:** chronic stress, tumor microenvironment, immunotherapy, anticancer treatments, sympathetic nervous system, neural-immune interactions, HPA axis, personalized medicine

## Abstract

Chronic stress has a substantial influence on the tumor microenvironment (TME), leading to compromised effectiveness of anti-cancer therapies through diverse mechanisms. It disrupts vital functions of immune cells that play a critical role in anti-tumor immunity, such as the inhibition of dendritic cells (DCs) and lymphocytes, while simultaneously enhancing the activity of immune cells that support tumor growth, such as myeloid-derived suppressor cells and tumor-associated macrophages. Furthermore, chronic stress exerts a significant impact on crucial mechanisms within the TME, including angiogenesis, DNA repair, hypoxia, extracellular matrix deposition, and tumor metabolism. These alterations in the TME, induced by stress, result from the activation of the hypothalamic–pituitary–adrenal axis and sympathetic nervous system, in conjunction with epigenetic modifications. In conclusion, chronic stress significantly influences the TME and impedes the efficacy of anti-cancer treatments, underscoring the importance of targeting stress pathways to improve therapeutic results.

## 1 Introduction

Chronic stress has been identified as a significant factor in the initiation and advancement of tumors, as it fosters inflammation and leads to adverse cancer-related consequences such as depression, fatigue, sleep disturbances, and unfavorable prognosis (Kruk et al., 2019; Russell and Lightman, 2019; Conceição et al., 2021). This phenomenon can be ascribed to the enduring stimulation of the hypothalamic–pituitary–adrenal (HPA) axis and the sympathetic nervous system (SNS) during prolonged episodes of chronic stress. Consequently, stress hormones such as catecholamines [including norepinephrine (NE) and epinephrine (E)] are discharged from sympathetic nerves, while cortisol is liberated from the adrenal cortex (Wang et al., 2022; Wu et al., 2022b). In cancer patients during diagnosis and treatment, various neuroendocrine factors such as dopamine, prolactin, nerve growth factor, BDNF, substance P, and oxytocin are commonly observed and undergo central regulation in response to stress (McEwen and Boyd, 2018; Mravec et al., 2020). These neurotransmitters possess the ability to impact immune and endothelial cells within the TME, thereby facilitating tumor progression (Jiang et al., 2020; Battaglin et al., 2022). The nervous and immune systems establish communication through shared soluble mediators and receptors, enabling the brain to detect inflammation and modulate the immune response (Dantzer, 2018). The SNS exerts a dual effect on the immune system, enhancing humoral immune responses while concurrently suppressing cell-mediated immune responses through the inhibition of cytotoxic activity in T lymphocytes and NK cells. This ultimately results in an immunosuppressive TME mediated by stress hormones, particularly glucocorticoids (Qin et al., 2021).

The TME has gained recognition as a crucial element in the pathogenesis of cancer, encompassing stromal cells, immune cells, endothelial cells, and other resident cell types. These cells, previously considered passive observers in tumor development, are now acknowledged to have a pivotal role in driving cancer progression. A multitude of factors, encompassing intrinsic attributes of cancer cells, the location of tumor formation, the stage of the tumor, and variables specific to the patient, exert an influence on the composition and functional state of the TME, resulting in notable variations (Winkler et al., 2023). The dynamic interplay between cancer cells and the TME, comprising stromal cells and extracellular matrix constituents, plays a crucial role in fostering cancer cell heterogeneity, clonal evolution, and the development of multidrug resistance. Ultimately, these interactions expedite the advancement and dissemination of cancer (Kamiya et al., 2021).

Chronic stress has been shown to affect the effectiveness of anticancer treatments by exerting an influence on the TME (Hong et al., 2022). The SNS plays a role in the formation of pre-metastatic niches (Ieguchi et al., 2022). Neurological signals and pathways have an impact on various cancer characteristics, such as metabolism and (epi)genomic stability, as well as supporting microenvironments that promote tumor growth, including immune infiltration and the extracellular matrix (Vaes et al., 2022). Numerous studies have provided evidence that chronic stress can induce alterations in the TME, affecting tumor cells, cancer stromal cells, and the extracellular matrix, thereby facilitating the progression of cancer.

This article provides an overview of the impact of chronic stress on the TME, elucidates the underlying mechanisms responsible for TME alterations induced by chronic stress, and underscores the detrimental consequences of chronic stress on anticancer treatment efficacy. These findings underscore the imperative for the implementation of comprehensive anticancer strategies.

## 2 Mechanisms of TME alterations under chronic stress

Chronic stress significantly impacts the tumor microenvironment through two primary mechanisms: the activation of the HPA axis and the SNS. The HPA axis involves the secretion of CRH and adrenocorticotropic hormone (ACTH), which then triggers the release of cortisol by the adrenal gland. Similarly, the SNS can stimulate the secretion of E and NE by the adrenal medulla or neurons.

**Figure 1:**
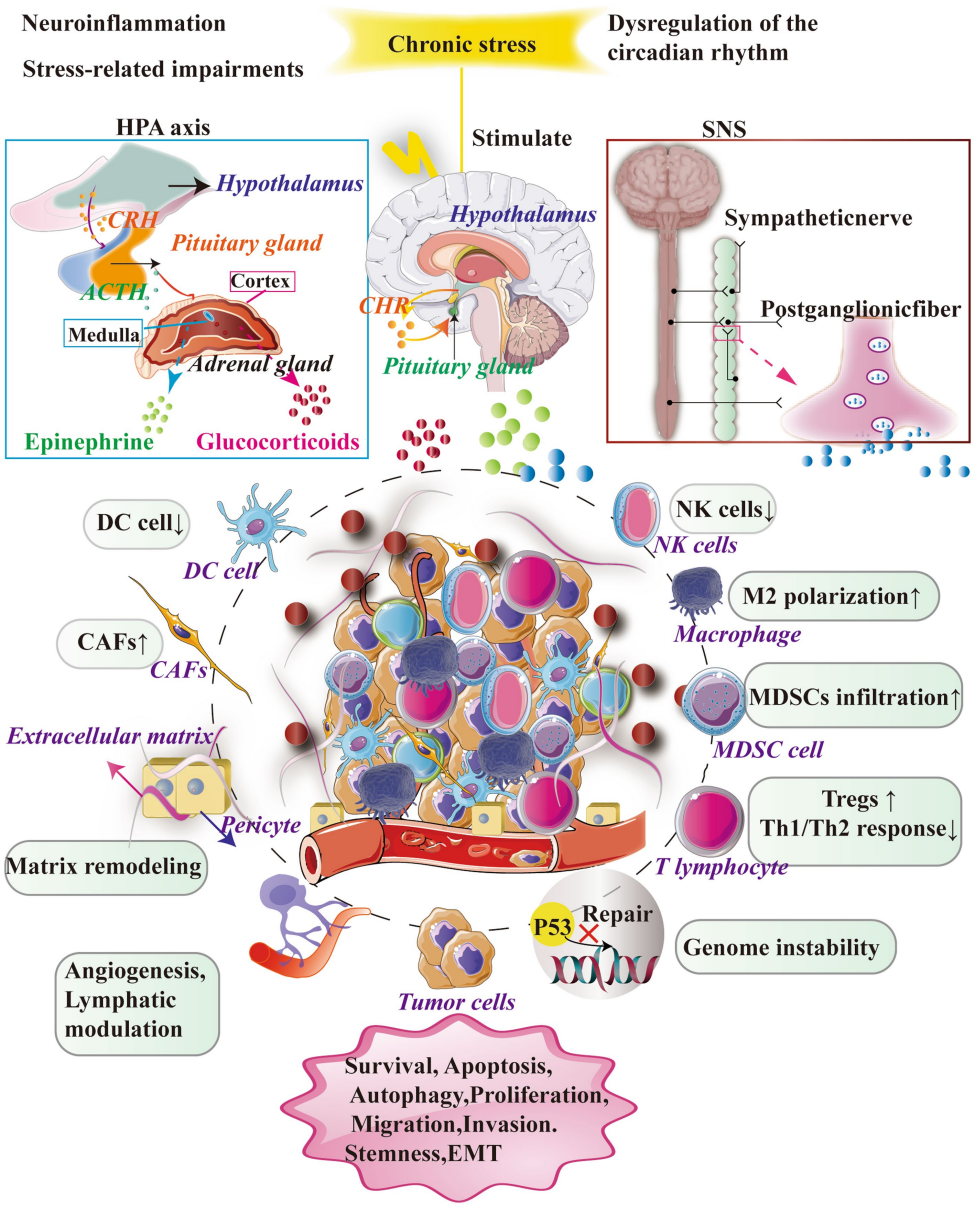
Chronic stress exerts a significant influence on the TME by primarily activating essential neuroendocrine response systems, namely the SNS and the HPA axis. The activation of the SNS leads to the secretion of NE and facilitates the synthesis and release of adrenaline and noradrenaline. However, the activation of the HPA axis results in the release of CRH from the hypothalamus, which in turn stimulates the secretion of adrenocorticotropic hormone ACTH and glucocorticoids (GCs) from the pituitary gland. It is worth noting that this complex cascade plays a significant role in facilitating the secretion and release of GCs from the adrenal cortex. Furthermore, the impact of stress-related hormones on cancer cells is mediated through their interaction with receptors on immune cells, thereby facilitating crucial biological processes including cell survival, programmed cell death, cellular self-degradation, cell division, cellular movement, and infiltration.

When triggered by chronic stress, these neuroendocrine systems exert diverse effects on immune and tumor cells within the tumor microenvironment. Chronic stress alters the composition and status of immune cells, impairs immune responses, and enables tumor cells to circumvent immune surveillance and develop resistance to drugs. Furthermore, chronic stress disrupts crucial processes such as angiogenesis, cell apoptosis, and metabolism, ultimately fostering tumor growth and progression (Figure 1; Tian et al., 2021).

### 2.1 Immune cells

Extended periods of stress can hinder the immune system’s capacity to safeguard the body through the suppression of T cells, natural killer cells, and macrophages (Antoni and Dhabhar, 2019; Wieduwild et al., 2020; Thapa and Cao, 2023). Furthermore, chronic stress can enhance the accumulation of immune-inhibitory cells, specifically regulatory T and B cells, as well as tumor-associated macrophages. Additionally, persistent stress triggers inflammation in the TME, facilitating reciprocal interactions between cancerous and inflammatory immune cells. Upon infiltration into the TME, immune cells undergo metabolic adaptations (Bondar and Medzhitov, 2013; Bader et al., 2020). Tumor-associated macrophages (TAMs), tumor-infiltrating lymphocytes (TILs), DCs, and myeloid-derived suppressor cells (MDSCs) employ paracrine signaling to regulate cancer growth by releasing pro-inflammatory cytokines. These cellular components establish communication through direct interactions and the secretion of cytokines and chemokines, utilizing autocrine and paracrine mechanisms (Lorton and Bellinger, 2015; Chen and Mellman, 2017; Greten and Grivennikov, 2019).

#### 2.1.1 Dendritic cells

DCs are essential in initiating adaptive immunity against cancer through the presentation of tumor antigens (Gardner and Ruffell, 2016; Sommershof et al., 2017). Nevertheless, chronic stress can undermine their functionality and result in immune suppression among recipients of cancer vaccines. This impairment is associated with the increased expression of stress-induced hormones and Tsc22d3, which diminish dendritic cell maturation and hinder their capacity to activate TCD8^+^ cells. To address this issue, reactivating the β2-AR receptor pathway and increasing Phosphorylated cAMP response element binding protein (pCREB) levels is crucial to restore CD40 signal transduction in DCs (Singh and Ranjan, 2023), and enhance the effectiveness of basic dendritic cell-based cancer vaccine therapy (Yang et al., 2019).

#### 2.1.2 Lymphocytes/tumor-infiltrating lymphocytes

Chronic stress harms supportive T cells and increases immune-suppressive T cells, especially in breast cancer patients (Kamiya et al., 2019). Stress also upregulates TIGIT, an immune receptor critical in regulating anti-tumor and antiviral immune responses (Rudak et al., 2019). However, stress has been found to have a preserving effect on mucosal-associated invariant T cells, while simultaneously impairing their TH1/TH2-type responses (Rudak et al., 2021). T cells are typically categorized based on central markers such as CD8 and CD4, as well as receptor subunits (Paijens et al., 2021).

#### 2.1.3 CD8 ^+^t

The exploration of signaling mechanisms that influence the phenotype of CD8^+^ T cells within the TME carries substantial implications, as it offers valuable insights for the advancement of novel approaches in cancer treatment. β-adrenergic receptors (β-ARs) exert a significant regulatory function through the modulation of immune checkpoint molecules, resulting in the suppression of anti-tumor immune responses and the simultaneous upregulation of PD-1, TIM-3, and Lag3, ultimately leading to T cell exhaustion (Nissen et al., 2018; Acharya et al., 2020). Additionally, the endogenous signaling of glucocorticoids within the TME impacts the differentiation of CD8^+^T cells, thereby compromising the effectiveness of immune checkpoint blockade (Qiao et al., 2021). Activation of the SNS via β-ARs has been observed to modulate the metabolism of CD8^+^ T cells within the TME, resulting in hindered infiltration of these cells in murine models of melanoma and colon cancer (Geng et al., 2023). Moreover, the inhibition of CD8^+^ T cell infiltration and function caused by NE has been found to confer resistance to anti-PD-1 monoclonal antibodies in cases of lung adenocarcinoma and gastric cancer (Muthuswamy et al., 2017; Vaes et al., 2022). Additionally, the release of calcitonin gene-related peptide (CGRP) by tumor-associated neurons has been directly implicated in the exhaustion of CD8^+^ T cells, thereby impairing their ability to eliminate melanoma cells (Balood et al., 2022).

#### 2.1.4 CD4 ^+^t

CD4 ^+^T cells are essential components of the anti-tumor immune response as they stimulate CD8^+^ T cells and induce DCs to express CD40 ligand (Borst et al., 2018). The differentiation of CD4 ^+^T cells is influenced by specific combinations of cytokines, which can also lead to the generation of regulatory Tregs. Tregs have been linked to tumor growth due to their ability to produce immunosuppressive cytokines (Knochelmann et al., 2018). The glucocorticoid-induced leucine zipper (GILZ) plays a crucial role in promoting the development of regulatory T cells (Tregs) by inducing the expression of FoxP3. Furthermore, the activation of the β2-AR in Tregs impedes their ability to produce inhibitory cytokines (Cannarile et al., 2019). However, the presence of chronic stress has been observed to result in an elevation of CD4^+^T cells within tumor tissues, thereby potentially facilitating the differentiation of cancer stem cells (Thapa and Cao, 2023). According to T-cell-extrinsic mechanisms, stress hormones cause CD4^+^T cells to differentiate toward a Th2 response (Capelle et al., 2022). The utilization of αGITR antibodies to mitigate Tregs functionality has been discovered to induce the differentiation of CD4^+^T cells into effector T cells, diminish Tregs-mediated immune suppression, and generate anti-tumor impacts (Zenga et al., 2022). The potential alleviation of certain negative effects of chronic stress can be achieved through β-ARs antagonism (Partecke et al., 2016).

#### 2.1.5 Natural killer cells

The role of NK cells in preventing and treating infections and cancer has long been recognized (Myers and Miller, 2021). Adrenaline and noradrenaline inhibit DNA-dependent mechanisms and protein synthesis, resulting in a reduction in cytokine production, including IL-2 and IFN-γ, and IL-12 (Neeman and Ben-Eliyahu, 2013). In addition to its role in regulating humoral immune responses, the SNS also plays a role in limiting the cytotoxic functions of T lymphocytes and NK cells in cellular responses (Jiang et al., 2017). The increased expression of programmed cell death receptor-1 (PD-1) in tumor-infiltrating NK cells, induced by glucocorticoid-mediated signaling, ultimately contributes to the progression of hepatocellular carcinoma in individuals with depression (Zhao et al., 2019).

#### 2.1.6 Myeloid-derived suppressor cells

The immunosuppressive effects of cancer therapy involve the active involvement of MDSCs in mediating immune suppression (Bruno et al., 2019). MDSCs represent a specific subset of immature myeloid cells (Iñigo-Marco and Alonso, 2019). The process of chronic stress has been observed to enhance the accumulation of MDSCs via the IL-6/STAT3 signaling pathway, thereby creating a conducive environment for metastasis (Cheng et al., 2019; Mohammadpour et al., 2019; An et al., 2021). Additionally, chronic pressure-induced adrenal signals effectively stimulate the proliferation and activation of MDSCs, leading to the establishment of an immunosuppressive microenvironment (Mohammadpour et al., 2021). The activation of the CXCL5-CXCR2-Erk cascade, induced by chronic restraint stress, plays a crucial role in driving the regulatory and recruiting functions of MDSCs (Cao et al., 2021). Furthermore, it is important to note that psychological stress serves as a catalyst for the activation of MDSCs within the spleen via TAM/CXCL1 signaling. This process plays a pivotal role in facilitating the development of pre-metastatic niches in breast cancer (Zheng et al., 2023).

#### 2.1.7 Macrophages

Additionally, macrophages play a vital role in modulating the TME, thereby influencing various aspects such as angiogenesis, remodeling of the extracellular matrix, proliferation of cancer cells, metastasis, immune suppression, and resistance to treatment (DeNardo and Ruffell, 2019; Mantovani et al., 2022). Psychological depression has been observed to exert a notable influence on the infiltration of macrophages in tumors, thereby significantly modulating their activity and inhibitory effects, ultimately impacting prognosis and treatment outcomes (Cassetta and Pollard, 2018, 2023). It has been found that the exposure of tumor cells to NE elicits the secretion of neuropeptide Y (NPY), which in turn facilitates the recruitment of macrophages and subsequent release of interleukin 6 (IL6). This phenomenon leads to the activation of the STAT3 signaling pathway in prostate cancer cells (Cheng et al., 2019). Furthermore, NE exerts control over the CCL2/CCR2 pathway through the stimulation of the β-ARs, thereby facilitating the migration and colonization of tumor cells in the lungs prior to metastasis (Chen et al., 2018). Moreover, the β3-AR receptor plays a crucial role in maintaining the equilibrium between M1/M2 macrophages and N1 granulocytes within the TME (Calvani et al., 2019). Glucocorticoids (GC) disrupt the ability of macrophages to effectively eliminate tumor cells by modulating the equilibrium between the “eat me” signal receptor (LRP1) and the “do not eat me” signal receptor (SIRPα) (Wu et al., 2022a). Additionally, the SNS expedites the activation of Kupffer cells via the ADRα1 receptor, thereby maintaining an inflammatory microenvironment that facilitates the progression of liver cancer (Huan et al., 2017).

#### 2.1.8 Cancer-associated fibroblasts

CAFs play a pivotal and multifaceted role in the TME, influencing various aspects such as tumor cell survival, metastasis, angiogenesis, immunosuppression, and resistance to therapeutic interventions (Zhang Y. et al., 2023). Empirical evidence substantiates the hypothesis that the commencement of α2-AR acts as a trigger for the proliferation and dissemination of CAFs, thus leading to an augmentation of the concentration of TGF-β within the confined TME (Shan et al., 2014). These signaling molecules effectively facilitate the division of tumor cells and the formation of new blood vessels, thereby promoting the supply of essential nutrients and oxygen to sustain tumor growth (Chen and Song, 2019; Bejarano et al., 2021; Rimal et al., 2022). Additionally, catecholamines, such as NE, proficiently regulate CAFs by activating β-ARs, thereby inducing heightened collagen synthesis. The presence of reinforced structural support within the tumor tissue facilitates the migration, invasion, and metastatic progression of tumor cells to distant sites (Nagaraja et al., 2021).

### 2.2 Cytokines

Scientific evidence unequivocally links cancer progression with the presence of inflammatory mediators like TNF-α, IL-6, TGF-β, and IL-10 (Liu N. et al., 2021). This correlation has intricated and widespread effects, as chronic stress disrupts cytokine secretion and activates multiple cellular pathways that significantly impact the occurrence and progression of malignant tumors. Furthermore, chronic stress actively promotes tumor development by affecting specific cellular pathways (Märkl et al., 2022; Vignjević Petrinović et al., 2023). For instance, the CXCL3-mediated Wnt/β-catenin pathway alters fatty acid metabolism (Lou et al., 2023), while dopamine-induced nuclear translocation of DRD2 impairs HIF1α degradation (Bernabé, 2021), thus promoting malignant cancer progression. Notably, chronic restraint stress triggers hepatocellular carcinoma growth via the β-ARs signaling pathway, and acute myeloid leukemia is linked to the HMGB1/NLRP3/IL-1β signaling pathway (Liu N. et al., 2021). Interestingly, rapid activation of β2-ARs skillfully regulates inflammation by inducing IL-10 secretion (Ağaç et al., 2018). However, this activation also has the potential to exacerbate cytokine and chemokine secretion, thereby compromising immune cell functionality (Thapa and Cao, 2023).

### 2.3 Angiogenesis

Additionally, sympathetic NE has been shown to promote tumor progression and angiogenesis through interaction with β-ARs in endothelial cells (Hondermarck and Jobling, 2018; Zhang et al., 2019; Cervantes-Villagrana et al., 2020; March et al., 2020). Chronic stress has been found to have a detrimental effect on cancer metastasis and the expression of various factors such as VEGF, MMP-2, MMP-7, and MMP-9 (Harjes, 2017; Stone, 2018). The activation of β2-ARs signaling has been shown to inhibit PPARγ activity, thereby promoting the growth of breast cancer and VEGF/FGF2-mediated angiogenesis (Zahalka et al., 2017; Zhou et al., 2020; Jeong et al., 2022). Additionally, β- ARs signaling has been found to suppress HDAC2, contributing to tumor angiogenesis and the progression of prostate cancer (Hulsurkar et al., 2017). Lastly, chronic stress has been observed to enhance angiogenesis through the activation of the PlexinA1/VEGFR2-JAK2-STAT3 pathway (Lu et al., 2021).

### 2.4 DNA damage

It is important to note that chronic stress has a significant impact on DNA integrity, regardless of the presence of carcinogens (Eckerling et al., 2021; Valente et al., 2021). Stress hormones are integral to the process of DNA damage repair, concurrently producing reactive oxygen species (ROS) and reactive nitrogen species (RNS) (Iftikhar et al., 2021). It is worth noting that adrenaline can induce DNA damage even in ovarian cancer cells (Lamboy-Caraballo et al., 2020). Moreover, prolonged stress reduces antioxidant activity, resulting in the buildup of free radicals, impeding DNA damage repair, and fostering the progression of skin cancer (Muqbil et al., 2020).

### 2.5 EMT

The chronic presence of stress facilitates tumor metastasis and invasion through the activation of diverse pathways (Blaes et al., 2020). For example, the miR-337-3p/STAT3 pathway has been observed to stimulate EMT in breast cancer cells and lung adenocarcinoma cells, thereby promoting invasion and migration (Du et al., 2020; Jiao et al., 2023). The activation of B2AR, MOR, and GSK3 by adrenaline and opioid drugs has been identified as a contributing factor to drug resistance, particularly in triple-negative breast cancer cases (Rousseau et al., 2022). EMT induction in both tongue squamous cell carcinoma and colorectal cancer is facilitated by the activation of distinct signaling pathways (Liu et al., 2018; Zhou et al., 2022). The impact of the β2-AR agonist salbutamol on EMT, migration, and invasion has been substantiated through the activation of the ERK phosphorylation pathway (Lu et al., 2022). Additionally, melatonin has been proven to inhibit the metastasis of epithelial ovarian cancer induced by chronic stress by modulating the NE/AKT/β-catenin/SLUG axis (Bu et al., 2020).

### 2.6 Extracellular matrix

ECM plays a pivotal role in tumors, providing essential functions such as structural support, regulation of the microenvironment, and provision of signaling molecules (Huang et al., 2021). The continuous activation of FAK, which is facilitated by the chronic stress-induced signaling transduction of β-ARs through the cAMP/PKA pathway, initiates a consequential cascade in which Erk1/2-MMP orchestrates the remodeling of the ECM (Cheng et al., 2018). Importantly, this remodeling phenomenon plays a significant role in enhancing invasive properties and metastatic potential in the pathogenesis of melanoma and prostate cancer (Ji et al., 2019).

### 2.7 Metabolic disorder

Furthermore, chronic stress exerts a profound influence on the immune system, leading to disruptions in mitochondrial function and cellular metabolism (Fan et al., 2019; Ruan et al., 2021). It serves as a potent stimulus for the activation of specific adrenaline receptors (β1ARs and β3ARs), initiating a series of events that result in the accumulation of lipid droplets in MCF-7 breast cancer cells. This accumulation disrupts the normal metabolic processes to a significant extent (Silva et al., 2023). The activation of β-ARs additionally alters cellular metabolism, hindering oxidative phosphorylation and encouraging angiogenesis in prostate cancer (Faulkner et al., 2019). Importantly, prolonged stress stimulates LDHA-dependent glucose metabolism via USP28, thereby enhancing the stability of MYC (Cui et al., 2019).

### 2.8 Hypoxia

Pressure plays a crucial role in regulating the function of immune cells by influencing tissue perfusion (He et al., 2022). The activation of sympathetic nerves or the administration of adrenergic receptor agonists induces a state of tumor hypoxia within the TME, which impedes the migration of leukocytes and promotes tumor progression. The simultaneous activation of the α1d-AR by chronic stress and *Helicobacter pylori* infection initiates the ubiquitination process of the SerpinA1 complex, leading to hypoxia and facilitating its interaction with IL-1α. This interaction ultimately promotes the development of gastric tumors (Devi et al., 2021; Aziz et al., 2022; Sullivan et al., 2023).

## 3 The chronic stress affects the efficacy of cancer treatment

The effectiveness of cancer treatments is negatively impacted by chronic stress (Wu et al., 2022b; Table 1).

**Table 1:** Chronic stress impairs cancer treatment efficacy.

Stressor	Target	Cancer type	References	Mechanism
β2-AR	CD8^+^ T cells	Melanoma	Qiao et al. (2019)	Metabolic reprogramming inhibition
GR	CD8^+^ T cells	Pancreatic cancer	Deng et al. (2021)	Activate PD-L1 expression
GR	DCs	NSCLC	Yang et al. (2019)	Block IFNs and IFN-γ activation
Corticosteroids	T-cell	NSCLC	Arbour et al. (2018)	PDL1 blockade efficacy decreased
GR	T, B, NK cells	MPM, gastric cancer, NSCLC, HCC, esophagus cancer	Aston et al. (2019) and Cui Y. et al. (2023)	Increases PD-1 expression level
GR	CD8^+^ T cells	Melanoma	Acharya et al. (2020)	Promotes dysfunction
β-ARs	Macrophages	Pheochromocytoma	van der Heijden et al. (2020)	Boosting glycolysis and oxidative phosphorylation
β1-AR	CD8^+^ T cells	Melanoma, pancreatic cancer	Globig et al. (2023)	CD8 T cell exhaustion
β2-AR	MDSC	Breast cancer	Mohammadpour et al. (2021)	Autophagy-driven boost in PGE2 production
β2-AR	CD8^+^ T cells	Lung adenocarcinoma	Geng et al. (2023)	Suppression of chemotaxis
GABA	B, T cells, TAM	Colon carcinoma	Zhang et al. (2021)	Elicits IL-10 macrophages
GR	DCs, CD8^+^ T cells,	Melanoma	Sommershof et al. (2017)	Exhaustion of CD8 T cells
GR	MDSC	Breast cancer	Zheng et al. (2023)	Enhance TAM/CXCL1 signaling
β2-AR	DCs	Neck tumors	Singh and Ranjan (2023)	Reduce CD40 expression on DCs
GR	T cells	Thymoma	Rudak et al. (2019)	T cell enhanced expression of TIGIT
GR	NK, T cells	Melanoma	Rudak et al. (2021)	Regulates TH1 and TH2 responses
β-ARs	CD8^+^ T cells	B-cell lymphoma	Nissen et al. (2018)	Minimize proliferation of T cells
β2-AR	CD8^+^ T cells	Melanoma	Qiao et al. (2021)	CD8 T cell exhaustion
β-ARs	Macrophage	Breast cancer, colon carcinoma	Muthuswamy et al. (2017)	Boost COX-2, IDO, IL-10, IFN
CGRP	CD8^+^ T cells	Melanoma	Balood et al. (2022)	Exhaustion of CD8 T cells
CGRP	CD4^+^ T, CD8^+^ T, NK cells,	HNSCC	McIlvried et al. (2022)	NK cells and CD4+ T cells are exhausted
β2-AR	CD4^+^T cells	HNC	Zenga et al. (2022)	Inducing CD4+ T cells to secrete SDF-1
β-ARs	TAM, CD8^+^ T, MDSCs	HCC	Liu C. et al. (2021)	β-ARs/CCL2/ PD-1
β2-AR	Metabolism	Colorectal cancer	Guan et al. (2023)	Activate PKA/CREB1 to enhance glycolysis
β1-AR	Tregs	HCC	Fu et al. (2023)	Diversifying immunosuppressive MAIT cells
β2-AR	Angiogenesis	Melanoma	Schuster et al. (2023)	Increased expression of VEGF-A, COX2, and IL6
β-ARs	MDSC	HCC	Cao et al. (2021)	CXCL5-CXCR2-Erk pathway activation
β-ARs	CD4^+^T cells, tregs	Pancreatic cancer	Partecke et al. (2016)	CTLA-4 reduction in CD4 cells
β-ARs	MDSCs	Breast cancer	An et al. (2021)	Activate IL-6/STAT3 signaling
β2-AR	MDSCs	Breast cancer	Mohammadpour et al. (2019)	Fas–FasL interact when STAT3 is activated
NPY	TAM, MDSCs	Prostate cancer	Cheng et al. (2019)	Activate IL6-STAT3
β3 -AR	Tregs, MDSCs, TAM	Melanoma	Calvani et al. (2019)	Reduced N1 granulocytes and M1/M2 macrophages
α1-ARs	TAM	HCC	Huan et al. (2017)	IL-6 and TGF-β are upregulated
β2-AR	EMT	Gastric adenocarcinoma	Shan et al. (2014)	Activation HIF-1α-Snail axis
β2-AR	CAFs	Ovarian cancer, breast cancer	Nagaraja et al. (2021)	Activate the β 2-AR/CREB/INHBA axis
β2-AR	Angiogenesis	Breast cancer	Zhou et al. (2020)	Angiogenesis induced by VEGF/FGF2
β2-AR	DNA damage	Ovarian cancer	Kang et al. (2016)	The expression of DUSP1 is elevated
β2-AR	CD8^+^ T cells	Melanoma	Chen et al. (2020)	Exhaustion of CD8 T cells
β2-AR	EMT	Lung cancer	Zhang X. et al. (2020)	Enhancing expression of Wnt1 and Drosha
β2-AR	T cells	Cancer	Farooq et al. (2023)	CAR-T therapy to fail Causing
β2-ARs	CD8^+^ T cells	Cancer	Daher et al. (2019)	CD8 T cell exhaustion
α1B, α1D, β2-AR	DNA damage	NSCLC	Xie et al. (2019)	Promote Cx32 expression for EGFR-TKI resistance.
Ach	CD8^+^ T cells	Thyroid cancer	Wang et al. (2020)	Activate the CD133-Akt pathway
Ach	EMT	SCC	Kwok et al. (2021)	Activate the nAChRα1/STAT7/NRF3 pathway
β3-AR	Tregs, MDSCs	NB	Bruno et al. (2023)	Increase IFN-γ and PD-L1 expression

GR, Glucocorticoid receptor; CGRP, Calcitonin gene-related peptide; NPY, Neuropeptide Y; MPM, Malignant pleural mesothelioma; HNC, Head and neck cancer; HNSCC, Head and neck squamous cell carcinoma; SCC, Squamous cell carcinoma; NB, Neuroblastoma, MAIT, Mucosa-associated invariant T.

### 3.1 Chemotherapy

Chronic stress disrupts the cellular response to DNA damage, thereby exerting detrimental effects on chemotherapy. It is worth noting that NE not only influences the resistance of ovarian cancer cells to cisplatin but also compromises DNA integrity (Lamboy-Caraballo et al., 2020).

Furthermore, chronic stress weakens the apoptotic response of tumor cells triggered by chemotherapy, ultimately impairing the efficacy of cytotoxic agents. This impairment is exacerbated by stress-induced glucocorticoids, which increase cancer cell resistance and reduce the efficacy of multiple chemotherapy drugs, such as paclitaxel, doxorubicin, TRAIL, and fluorouracil (Kanai et al., 2020; Cui et al., 2021; Butz and Patócs, 2022; Mitre-Aguilar et al., 2022). Additionally, NE inhibits c-Jun phosphorylation, mediated by JNK, through activation of the β 2-AR pathway, thereby attenuating the apoptosis of ovarian cancer cells induced by paclitaxel (Kang et al., 2016; Chen et al., 2021).

### 3.2 Radiotherapy

Radiation oncology is primarily responsible for the occurrence of distant effects that can result in long-lasting damage to nearby radiation-sensitive nerves. The incidence of distant events following radiotherapy closely correlates with the level of adrenergic stress experienced by the host. Chronic stress has been found to have detrimental effects on T-cell function, gene expression, and signaling pathways, leading to a reduction in immune factor secretion. Adrenergic agonists exacerbate immune suppression and promote the presence of M2 macrophages, further compromising the immune system’s ability to regulate tumor growth. Furthermore, adrenergic agonists also impede the activity and function of CD8 ^+^T cells, directly affecting their effectiveness in targeting tumors (Chen et al., 2020). Of particular significance is the decrease in expression of IFN-γ and Granzyme B in T cells caused by adrenergic agonists, which limits their effector phenotype. This reduction ultimately promotes resistance among tumor cells to radiation-induced cell death, thereby disrupting the anticancer effects of radiation (MacDonald et al., 2019). Additionally, β2-ARs agonists amplify the expression of Wnt1 and Drosha in LLC-1 cells, hindering radiation-induced cell apoptosis (Zhang X. et al., 2020).

### 3.3 Immunotherapy

Extensive preclinical research has supported the adverse effects of prolonged stress on the effectiveness of immune checkpoint inhibitors, tumor vaccines, and immune stimulants (Liu et al., 2023). It has also been demonstrated that psychological pressure reduces the therapeutic efficacy of IL-2 immunotherapy among patients with renal cell carcinoma, resulting in decreased expression of IL-2 receptors in peripheral blood leukocytes (Zhang L. et al., 2020). Additionally, scientists have observed that manipulation of glucocorticoid receptors in pancreatic cancer cells enhances immune evasion and resistance to immunotherapy, leading to changes in expression of PD-L1 and MHC-I genes (Deng et al., 2021). Furthermore, the β2-AR receptor has been found to disrupt the mitochondria’s adaptation of T-cells, impair T-cell activation, proliferation, cytokine release, and hinder the effectiveness of CAR-T cell therapy (Farooq et al., 2023). Also, chronic stress from social disruption suppresses CD8^+^ T-cell responses to cancer vaccines when administered via PLGA-MS, due to impaired dendritic cell maturation, migration, and CD8^+^ T-cell response initiation (Daher et al., 2019).

### 3.4 Molecularly targeted therapy

The research findings indicate that chronic stress impedes the inhibitory effects of EGFR, leading to a decrease in the effectiveness of the treatment. Additionally, the stress hormone NE increases the expression of Cx32 and enhances the transcription levels of proteins associated with resistance to EGFR-TKI, such as MET and IGF-1R (Xie et al., 2019). At the same time, it slows down the degradation rate of these proteins, further worsening the impact on the efficacy of Afatinib. Moreover, the activation of β2-AR has been found to inhibit LKB1 and promote the expression of IL-6, resulting in the development of resistance against EGFR TKI in non-small cell lung cancer (NSCLC) through the MAPK pathway (Nilsson et al., 2017; Chang et al., 2020).

## 4 Targeting chronic stress for enhanced anticancer therapies

**Figure 2:**
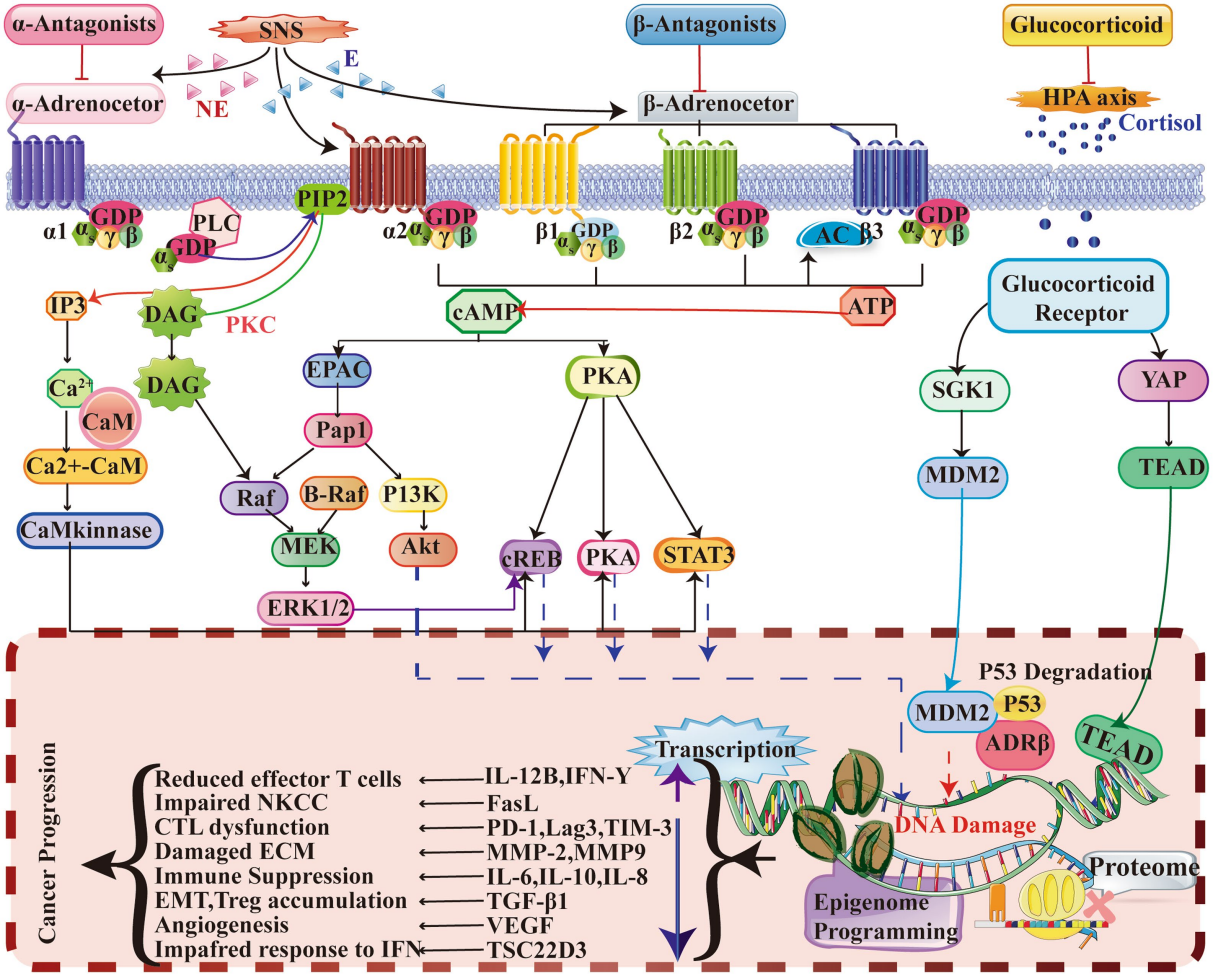
This study provides evidence that chronic stress induces the activation of a signaling pathway that significantly impacts the TME. ARs, specifically α-AR, β2-AR, and β3-AR, play a crucial role in this process. ARs function as G-protein coupled receptors (GPCRs) and bind to agonists such as adrenaline and noradrenaline. This binding activates intracellular Gαs protein, initiating a series of reactions that result in increased levels of intracellular IP3, DAG, and cAMP. Additionally, this activation simultaneously triggers second messenger pathways, including PKA, PKC, EPAC, and Ca^2+^-CaM. Furthermore, the involvement of glucocorticoid receptors (GRs), which are part of the nuclear receptor family, is also noteworthy. When GCs permeate the cell membrane and bind to GRs, the Hsp protein dissociates, enabling the primary subunit of GRs to relocate to the cell nucleus and initiate gene transcription. Consequently, the regulation of cytokine and ligand gene expression occurs, thereby impacting protein synthesis programming and epigenetic gene expression. Collectively, these signaling mechanisms induce changes that contribute to the deterioration of the TME and ultimately promote tumor development. E, Epinephrine; EPAC, Exchange protein directly activated by cAMP; DAG, Diacylglycerol; ERK, Extracellular signal-regulated kinase; PI3K, Phosphatidylinositol 3-kinase; Hsp., Heat shock protein; PKA, Protein kinase A; AC, Adenylyl cyclase; MDR, Multidrug resistance; GATA1, GATA-binding protein 1; STAT3, Signal transducer and activator of transcription 3; NE, Norepinephrine; CaM, Calmodulin; IP3, Inositol trisphosphate; PIP2, Phosphatidylinositol 4,5-bisphosphate; EMT, Epithelial-mesenchymal transition; PKC, Protein kinase C; PLC, Phospholipase C; CREB, cAMP response element-binding protein; GR, Glucocorticoid receptor; CTL, Cytotoxic T lymphocyte; ECM, Extracellular matrix; NKCC, NK cell cytotoxicity; IFN, Interferon; ATP, Adenosine triphosphate; cAMP, Cyclic adenosine monophosphate; MEK, Mitogen-activated protein kinase; Pap1, Pathway activator protein 1.

The significant influence of chronic stress on the initiation and progression of cancer has been consistently demonstrated in multiple studies. Therefore, it is imperative to prioritize targeted management of chronic stress in order to enhance the efficacy of anticancer therapies (Figure 2).

### 4.1 Medications targeting adrenergic receptors

The efficacy of ARs antagonists in the treatment of tumor formation and growth induced by chronic stress has been demonstrated (Nilsson et al., 2020). These antagonists encompass both alpha and beta blockers, as outlined in Table 2.

**Table 2:** Impact of targeted adrenergic receptor drugs on tumor growth.

Effect	Mechanisms	Targets	Drugs
BC, PC, GC	GPCR (Jabir et al., 2022)	α2-AR	Yohimbine, Rauwolscine
Renal cancer	Targeting AKT and FAK (Mihalopoulos et al., 2020)	α1-AR	Quinazoline
BC	Blocking ARs (Kamiya et al., 2019)	α-ARs	Phentolamine
BCa, PCa	Decreasing the expression of ELK1, C-FOS, NF-κB (Kawahara et al., 2016; Nagata et al., 2020)	α1a-AR	Silodosin
Glioma, OSCC	Upregulation of Autophagy (Liu B. et al., 2021; Liu Z. et al., 2021; Xing et al., 2023)	α1a-AR	Doxazosin
PCa, AML,	Inhibiting the PI3K/Akt/mTOR pathway (Li et al., 2020; Sun et al., 2020; Liu D. et al., 2021)	α1a-AR	Prazosin
PCa, BCa, carcinoma, OC, CCRCC	Regulation of Bcl-2 (Shimizu et al., 2020; Florent et al., 2020a,b; Zhong et al., 2021)	α1a-AR	Naproxen
Melanoma, NSCLC, BC	Boosting Caspase-3/7; ActivityFBXL2-Grp94-EGFR (Niu et al., 2021; Farhoumand et al., 2023; Li F. et al., 2023)	β1-AR	Nebivolol
Hepatoma, PCa, NSCLC	Inhibition of (FBXL10, TRAF6) (Springer et al., 2023; Yuan et al., 2023)	β1-AR	Bisoprolol
NSCLC	Inhibiting β1-AR (Jin et al., 2023)	β1-AR	Landiolol
NSCLC	Blocking ELK-1, AhR, NF-κB Activities (Shahid et al., 2023)	β1-AR	Carvedilol
Melanoma, CRC, BC, OC, PCa	Inhibiting AKT/MAPK; β-ARs and COX-2 Inhibition; CD8 T-cell Priming Suppression; IFN-γ and PD-L1 Reduction; Decrease EMT (Sorski et al., 2016; Shaashua et al., 2017; Haldar et al., 2018, 2020; Daher et al., 2019; Ricon et al., 2019; Hiller et al., 2020; Liao et al., 2020; Liang et al., 2021; Li et al., 2022; Cui Q. et al., 2023; Falcinelli et al., 2023)	β-ARs	Propranolol
GC, LUAD, melanoma, hepatoma	Suppression of the ERK1/2-JNK-MAPK pathway; β2-ARs/CCL2 axis (Zhang et al., 2019; Zhang B. et al., 2020; Liu C. et al., 2021; Ji et al., 2022)	β2-AR	ICI118551
BC, NB, melanoma	IFN-γ, PD-1/PD-L1; SK2/S1P2 Hemato-differentiation enhancement (Bruno et al., 2020; Calvani et al., 2020; Bruno et al., 2023)	β3-AR	SR59230A

ccRCC, Clear cell Renal Cell Carcinoma; OSCC, Oral Squamous Cell Carcinoma; AML, Acute Myeloid Leukemia; GPCR, G protein-coupled receptor; BC, Breast Cancer; PCa, Prostate Cancer; PC, Pancreatic Cancer; GC, Gastric Cancer; BCa, Bladder Cancer; OC, Ovarian Cancer; CRC, Colorectal Cancer; NSCLC, Non-Small Cell Lung Cancer; LUAD, lung adenocarcinoma; NB, Neuroblastoma.

#### 4.1.1 β-Blockers

The presence of catecholamines and β-ARs has been observed to exhibit correlation with various types of cancer, such as breast, bladder, glioma, prostate, colorectal, gastric, and melanoma cancers (Barathova et al., 2020; Matzner et al., 2020; Jayachandran et al., 2023; Li R.Q. et al., 2023). Increased sympathetic nervous system activity leading to elevated levels of catecholamines has been demonstrated to contribute to tumor growth, angiogenesis, and metastasis. Nevertheless, the mitigation of these carcinogenic effects can be achieved by inhibiting the interaction between catecholamines and β-ARs. For instance, research conducted on breast cancer cells subjected to the β-AR antagonist propranolol has revealed heightened levels of the tumor suppressor protein p53 and augmented cellular demise (Montoya et al., 2019). Propranolol has also exhibited efficacy in alleviating immune suppression facilitated by IFN-γ-induced PD-L1 expression in ovarian cancer (Falcinelli et al., 2023). Additional β1-AR blockers such as Nebivolol, Bisoprolol, and Landiolol have similarly exhibited potential in bolstering antitumor immune responses across various cancer categories, thereby potentially enhancing patient prognoses (Niu et al., 2021; Farhoumand et al., 2023; Jin et al., 2023; Li F. et al., 2023; Shahid et al., 2023; Springer et al., 2023; Yuan et al., 2023).

#### 4.1.2 α-Blockers

α-AR blockers, such as quinazoline, phentolamine, silodosin, doxazosin, prazosin, and naproxen, have demonstrated significant potential as effective cancer treatments (Suzuki et al., 2019). These medications have successfully impeded tumor growth and the development of new blood vessels in advanced prostate, bladder, and renal cancers. Silodosin, for instance, has been found to suppress the growth of prostate cancer cells by blocking α 1-AR. Similarly, doxazosin has shown the ability to hinder the multiplication of bladder cancer cells and restrict tumor expansion. The combination of these receptor blockers with traditional chemotherapy, radiotherapy, and anti-EGFR treatment has resulted in improved overall survival rates for prostate cancer patients (Ashrafi et al., 2017). Furthermore, they enhance autophagy, which facilitates the breakdown of damaged cellular components, thereby intensifying their anticancer effects (Archer et al., 2021). Additionally, α 1-AR blockers have the potential to mitigate adverse effects and enhance the efficacy of CAR-T cell therapy (Liu D. et al., 2021). Quinazoline and phentolamine have been found to alleviate symptoms of cytokine release syndrome in CAR-T cell therapy patients (Li et al., 2020; Sun et al., 2020; Liu D. et al., 2021). These findings emphasize the use of α-AR blockers as promising agents in cancer therapy, highlighting their capability to enhance treatment outcomes (Shimizu et al., 2020; Florent et al., 2020a,b; Zhong et al., 2021).

### 4.2 Psychosocial intervention for stress-related tumors

Interventions that directly target both physical and psychological stressors can effectively improve the TME, leading to enhanced efficacy of cancer treatments (Li et al., 2019; Tack et al., 2021, 2022). These interventions include carefully structured exercise programs (Zhang et al., 2016; Marker et al., 2018; McGettigan et al., 2020), the use of music therapy (Batty et al., 2017), dietary improvements (Goto et al., 2017; Jacka et al., 2017; Yang et al., 2020; Van Blarigan et al., 2023) and the establishment of healthier sleep patterns (Jacob et al., 2018; Chen Y.C. et al., 2022). It has been firmly established that chronic stress has a significant impact on the progression of cancer and is closely linked to the gut-brain axis (Jiang et al., 2023; Radford-Smith and Anthony, 2023). By regulating the gut microbiota, stress responses in individuals with tumors can be alleviated (Zhang Z. et al., 2020; Kumar et al., 2023; Li C. et al., 2023). A range of interventions, such as probiotics, prebiotics, postbiotics, and 5-HTP, can effectively achieve this outcome (Zhang Z. et al., 2020; Jiang et al., 2023; Kumar et al., 2023; Li C. et al., 2023; Radford-Smith and Anthony, 2023). Moreover, the incorporation of conventional, complementary, and integrative approaches, including acupuncture (von Trott et al., 2020; Han et al., 2021; Chen et al., 2023), meditation (Cramer et al., 2017; Kinkead et al., 2018), herbal medicine (e.g., Si-Ni-San) (Zhang J. et al., 2023) and Tai Chi (Irwin et al., 2017), has demonstrated efficacy in suppressing the proliferation and spread of tumors caused by chronic psychological stress.

### 4.3 Therapeutics for nerve fibers

The treatment of stress-induced tumors necessitates the implementation of antineuronal therapy and neural ablation (Magnon and Hondermarck, 2023). Neural ablation, which combines surgical techniques and pharmacological intervention, effectively alleviates symptoms associated with tumor growth (Zhao et al., 2014; Rabben et al., 2016). The inhibition of nerve growth factor (NGF) serves as a crucial approach to hinder the advancement of diseases by impeding the growth and restructuring of neural fibers linked to the tumor (Lin et al., 2023; Yan et al., 2023). Moreover, research has demonstrated a substantial association between the existence of nociceptor neurons and enhanced rates of survival among individuals afflicted with high-grade ovarian tumors (HGSOC) (Restaino et al., 2023). To summarize, interventions such as antineuronal therapy, neural ablation, and NGF inhibition play a significant role in the management of tumors induced by chronic stress (Logotheti et al., 2020).

### 4.4 Immune system regulation

**Figure 3:**
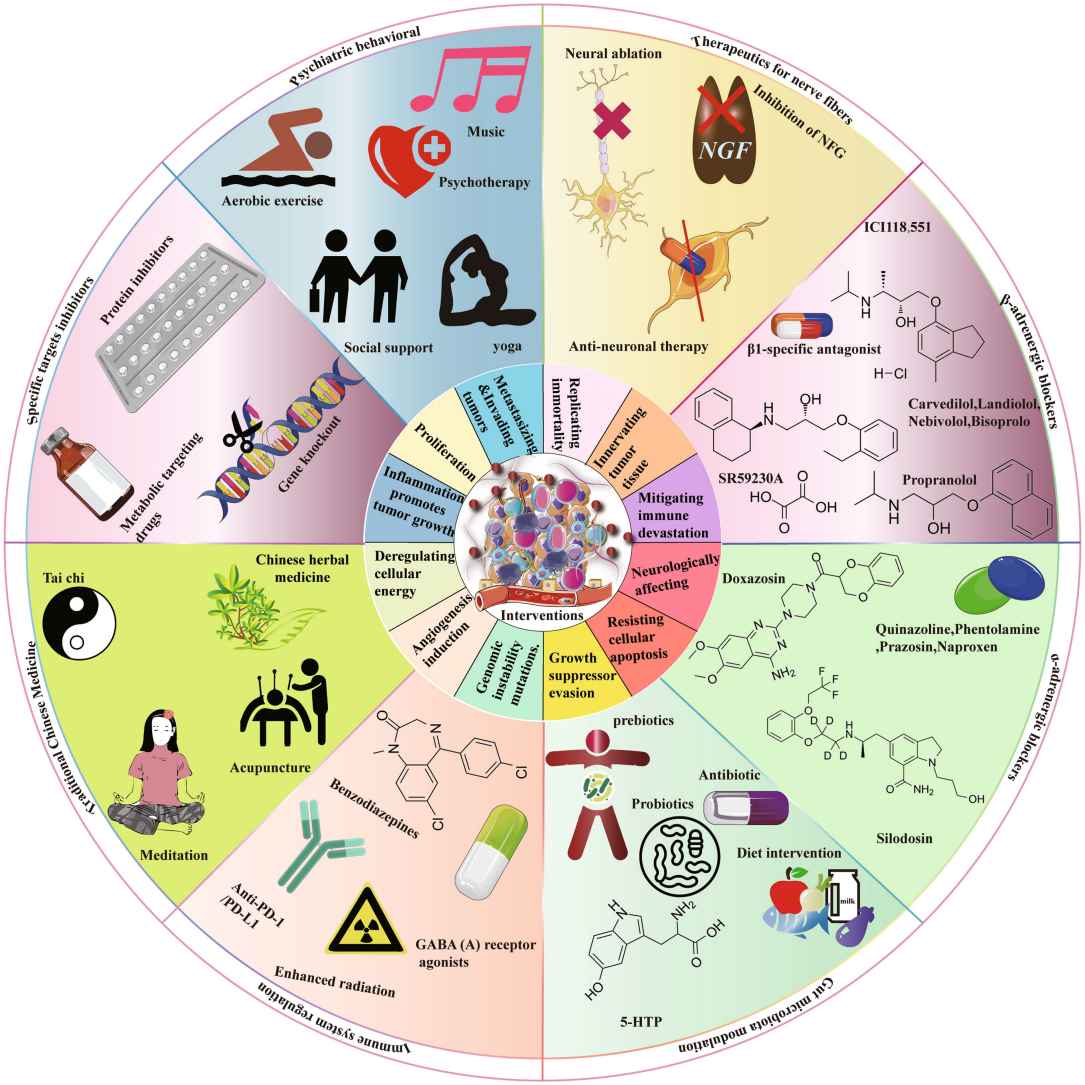
There are several strategies that can be implemented to mitigate the advancement of cancer resulting from chronic stress. These strategies encompass the use of targeted inhibitors, psychosocial interventions, neurofiber therapy, receptor blockers (including both β-ARs and α-ARs blockers), modulation of gut microbiota, modulation of the immune system, and the incorporation of traditional Chinese medicine. Targeted inhibitors encompass the utilization of pharmacological compounds, protein inhibitors, and other substances that specifically target metabolic processes. Psychosocial interventions encompass a diverse array of activities, including aerobic exercise, psychotherapy, yoga therapy, music therapy, and mindfulness training. Neurofiber therapy incorporates a multitude of treatment modalities, such as nerve ablation, inhibition of nerve growth factor, and interventions for anti-neural diseases. The classification of β-ARs blockers includes subcategories such as β2-AR blockers and β3-AR blockers, whereas α-ARs blockers encompass both α2-AR blockers and α1-AR blockers.

Targeting immune-suppressive neurotransmitters represents a potential approach for overcoming immune resistance and augmenting the anti-tumor immune response (Xiao et al., 2023). Notably, benzodiazepine-class drugs, functioning as GABA(A)R activators, have been observed to enhance the response to radiation and immune checkpoint blockade (ICB) by facilitating direct anti-tumor effects and the infiltration of CD8^+^ T cells. Additionally, dopamine signaling has demonstrated potential in promoting the differentiation of tissue-resident memory CD8^+^ T cells, thereby enhancing the effectiveness of chemotherapy in pancreatic cancer and regulating macrophage-mediated inflammation (Liu Q. et al., 2021; Chen Y. et al., 2022). The investigation of GITR in Tregs as a potential target has revealed that the use of antibodies can facilitate the transformation of Tregs into CD4 ^+^ T cells, thereby diminishing their inhibitory impact on tumor immunity (Amoozgar et al., 2021). Additionally, the administration of fluoxetine, an antidepressant, has been found to suppress the mechanisms induced by chronic stress in lung cancer, consequently augmenting the cellular immune response and leading to notable antitumor effects (Yang et al., 2021; Figure 3).

## 5 Conclusion

The examination of prolonged stress in cancer therapy holds significant significance due to its direct influence on the neoplastic microenvironment, ultimately compromising treatment effectiveness and impairing immune responses. It is imperative to acquire a comprehensive comprehension of the complex interaction between persistent stress, stromal cells, and the immune system to formulate effective treatment approaches. Additionally, elucidating the impact of prolonged stress on neoplastic genetics and expression patterns can yield valuable insights into treatment response and prognosis. The advent of innovative technologies, such as artificial intelligence and optogenetics, offers promising opportunities for addressing chronic stress and improving cancer therapies, thereby enabling personalized and targeted treatment strategies. Furthermore, the potential synergy between β-blockers and anti-angiogenic agents, immunotherapy, radiotherapy, or chemotherapy holds promise for enhancing treatment efficacy. The potential for alleviating the adverse impacts of prolonged stress lies in the implementation of mindfulness and meditation interventions, in conjunction with meticulous evaluation of drug dosage and timing. To advance cancer care and the field of personalized medicine, it is imperative to undertake a comprehensive examination of the interplay between persistent stress, the neoplastic microenvironment, stromal cells, the immune system, and treatment.

## Author contributions

YRL: Conceptualization, Methodology, Writing – original draft. FHL: Funding acquisition, Resources, Writing – original draft. YCT: Project administration, Writing – original draft. YNW: Formal analysis, Writing – original draft. FX: Supervision, Writing – review & editing. JHW: Funding acquisition, Validation, Writing – review & editing.
